# Population specific risk thresholds for lipoprotein(a): implications from angiographic data in Central Asia

**DOI:** 10.3389/fmed.2026.1749546

**Published:** 2026-04-23

**Authors:** Aisulu Mussagaliyeva, Rustem Tuleutayev, Sholpan Zhangelova, Dina Kapsultanova, Dana Akhmentayeva, Aruzhan Zhanuzak, Saltanat Altybayeva, Amina Rakisheva

**Affiliations:** 1 JSC “Research Institute of Cardiology and Internal Diseases”, Almaty, Kazakhstan; 2 Department of Internal Disease, Asfendiyarov Kazakh National Medical University, Almaty, Kazakhstan; 3 Qonaev City Hospital, Almaty, Kazakhstan; 4 International Medical School of the UIB, Almaty, Kazakhstan

**Keywords:** coronary angiogram (CAG), coronary artery disease, lipoprotein(a), low-density lipoprotein cholesterol, statin

## Abstract

**Background:**

Lipoprotein(a) [Lp(a)] is an established, genetically determined risk factor for atherosclerotic cardiovascular disease (ASCVD), but population-specific thresholds for cardiovascular risk remain uncertain. Data from Central Asia are particularly limited. We aimed to evaluate the relationship between Lp(a) concentrations and coronary artery disease (CAD) severity in a Kazakhstani cohort and to determine an Lp(a) threshold associated with obstructive coronary disease.

**Methods:**

In this single-center observational study, 238 adults (mean age 65.1 ± 0.6 years; 60% male) referred for elective coronary angiography were enrolled. Fasting venous blood samples were obtained for a full lipid panel and Lp(a) assessment. Lp(a) levels were quantified in nmol/L using an immunoturbidimetric method. CAD severity was categorized as no significant stenosis, 1-vessel, 2-vessel, or ≥3-vessel disease. Between-group comparisons were performed using t-tests and ANOVA. Receiver operating characteristic (ROC) analysis identified an optimal Lp(a) cut-off for predicting significant CAD (≥50% stenosis in ≥1 vessel), with the Youden index determining the threshold.

**Results:**

Significant CAD was present in 185 patients (78%), while 53 (22%) had no obstructive disease. Lp(a) levels increased stepwise with CAD severity: 36.5 ± 9.4 nmol/L (no stenosis), 45.3 ± 9.0 nmol/L (1-vessel), 76.7 ± 14.6 nmol/L (2-vessel), and 97.2 ± 15.8 nmol/L (≥3-vessel disease) (*p* < 0.05 for trend). Patients with prior myocardial infarction had significantly higher Lp(a) than those without (78.9 ± 11.9 vs. 51.9 ± 6.6 nmol/L, *p* = 0.04), whereas traditional lipid parameters did not differ by MI status or CAD extent. ROC analysis identified 37 nmol/L as the optimal threshold for predicting obstructive CAD. Patients with Lp(a) ≥ 37 nmol/L had markedly higher odds of significant stenosis (90% vs. 71%, OR 3.4, 95% CI 1.6–7.6, *p* < 0.001).

**Conclusion:**

In this Kazakhstani cohort, Lp(a) was strongly associated with both the presence and angiographic severity of coronary atherosclerosis, whereas LDL-C and other lipid parameters showed no such relationship. Clinically meaningful risk occurred at substantially lower Lp(a) levels than commonly used Western thresholds. These findings highlight the need for population-specific Lp(a) cut-offs in Central Asia and support routine Lp(a) measurement to improve cardiovascular risk stratification. Larger studies are warranted to refine regional thresholds and assess the value of emerging Lp(a)-lowering therapies.

## Introduction

Cardiovascular disease (CVD) remains the leading cause of mortality worldwide and in Kazakhstan. Despite advances in both primary and secondary prevention, adverse cardiovascular events continue to occur due to the so-called *residual cardiovascular risk* (1). This persistent risk has stimulated the search for additional, nontraditional risk factors and biomarkers that may refine risk prediction beyond conventional lipid parameters.

Among these, lipoprotein(a) [Lp(a)] has emerged as an independent and genetically determined risk factor for atherosclerotic cardiovascular disease (ASCVD), exerting its effects even in individuals with very low LDL-cholesterol levels (2). Lp(a) is an LDL-like particle containing apolipoprotein(a), which imparts pro-atherogenic, pro-inflammatory, and pro-thrombotic properties. Because Lp(a) concentrations are largely genetically determined (approximately 70–90% heritable) and minimally influenced by lifestyle or environmental factors, levels vary widely among individuals from birth (3, 4). Roughly one in five individuals (≈20%) inherit markedly elevated Lp(a) concentrations (>50 mg/dL or >105 nmol/L), conferring a substantially increased lifetime risk of CVD, whereas low Lp(a) levels (<30 mg/dL or ≈62 nmol/L) are associated with negligible vascular risk (5–7).

Notably, Lp(a) concentrations differ substantially across ethnic and geographic populations. For instance, data from the UK Biobank demonstrated median levels of 19 nmol/L in white Europeans, 31 nmol/L in South Asians, 75 nmol/L in individuals of African ancestry, and 16 nmol/L in Chinese participants (8, 9). Despite these absolute differences, the *relative* risk contribution of Lp(a) appears broadly consistent across populations. However, it remains uncertain whether a single universal threshold for “elevated” Lp(a) is appropriate for all ethnic groups. Most Western guidelines classify Lp(a) ≥ 50 mg/dL (≈105 nmol/L) as high-risk (10, 11), while lower thresholds (≈30 mg/dL) have been proposed for Chinese populations (11).

Central Asia represents a major gap in this evidence base. Data on Lp(a) distribution and clinical impact in countries such as Kazakhstan are extremely limited (12, 13). Kazakhstan’s multi-ethnic population—including Kazakh, Russian, Uyghur, and other groups—bears a high burden of premature CVD, yet no large-scale studies have characterized Lp(a) levels or their association with coronary atherosclerosis in this region. This lack of population-specific data hampers the development of locally relevant risk-stratification and prevention strategies.

To address this knowledge gap, we investigated the relationship between Lp(a) concentrations and the extent of coronary atherosclerosis in a cohort of Kazakhstani patients as such evidence may help clarify the prognostic value of Lp(a) in Central Asia and guide more personalized approaches to cardiovascular risk assessment and secondary prevention.

## Methods

### Study design and population

In this single-center, observational study, we hypothesized that higher lipoprotein(a) [Lp(a)] levels are associated with greater severity of coronary artery involvement and sought to identify a threshold level indicative of significant coronary artery lesions. Adults aged 18–85 years with a positive or inconclusive stress echocardiography or treadmill test, or with contraindications to functional testing, who were admitted for elective coronary angiography, were eligible for inclusion. Exclusion criteria included: hemodynamic instability (including patients receiving mechanical or pharmacological support with inotropic agents), recent acute coronary syndrome or cardiovascular procedure, severe renal impairment, history of clinically significant disorders including severe cardiac arrhythmias. All participants provided an informed written consent and the study protocol was approved by the local ethics committee.

### Lipid and Lp(a) measurements

Lp(a) concentrations were measured using an immunoturbidimetric assay calibrated in nmol/L, which is less affected by apolipoprotein(a) isoform size variability compared with mass-based assays. The assay was performed in an accredited laboratory following standardized quality control procedures. Calibration was conducted using manufacturer-provided reference materials traceable to international standards. LDL-C was directly measured following accredited laboratory protocols. Achievement of lipid targets was determined based on current ESC/EAS guideline thresholds for cardiovascular risk categories (e.g., LDL-C < 1.4 mmol/L for very-high-risk patients).

### Coronary angiography and assessment of CAD severity

Significant coronary stenosis during coronary angiography was defined as ≥50% luminal narrowing in any major epicardial coronary artery.

The extent of coronary artery disease (CAD) was categorized as follows:

No significant stenosis
Single-vessel disease (1 vessel)
Two-vessel disease (2 vessels)
Multivessel disease (≥3 vessels)

The presence or absence of any significant stenosis was recorded for each patient. Angiograms were interpreted by experienced operators who were blinded to patients’ Lp(a) concentrations.

### Statistical analysis

Patients were stratified according to Lp(a) levels for subgroup analyses. Continuous variables are presented as mean ± standard error of the mean (SEM), and categorical variables as counts and percentages.

Between-group comparisons (e.g., prior myocardial infarction (MI) vs. no MI) were performed using the independent Student’s t-test for continuous variables and the chi-square test for categorical variables. Analysis of variance (ANOVA) was applied to compare mean Lp(a) levels across CAD severity groups, followed by post-hoc testing where appropriate.

A *p*-value < 0.05 was considered statistically significant. To identify an Lp(a) threshold predictive of significant CAD, receiver operating characteristic (ROC) curve analysis was conducted. The Youden index determined the optimal cut-off maximizing sensitivity and specificity for detecting any significant coronary stenosis. The odds ratio (OR) for significant CAD associated with Lp(a) above versus below this threshold was then calculated, with 95% confidence intervals (CI).

### Ethical approval

The study was conducted in accordance with the principles of the Declaration of Helsinki and approved by the Institutional Ethics Committee. All patients provided informed consent prior to participation. Ethical approval was granted by the Asfendiyarov Kazakh National Medical University (No. 11(134) on 04 of November 2022).

## Results

### Study population

Baseline characteristics are summarized in Table 1. A total of 238 consecutive patients were enrolled over a ten-months period. The mean age was 65.1 ± 0.6 years, with 143 male (60%) and 95 female (40%). The study population consisted primarily of Kazakh participants (50.4%, *n* = 120), followed by Russians (28.6%, *n* = 68), Uyghur (10.5%, *n* = 25), and other ethnicities (10.5%, *n* = 25). The cohort was predominantly overweight (mean BMI 28.6 ± 0.3 kg/m^2^), with 33.6% (*n* = 80) classified as obese (BMI ≥ 30 kg/m^2^). Type 2 diabetes mellitus was present in 39.1% (*n* = 93). A prior MI was documented in 41.6% (*n* = 99), and 10.1% (*n* = 24) had a history of stroke. A positive family history of premature CVD was reported in 61% (*n* = 145). The mean age of CAD onset was 60.1 ± 0.6 years.

**Table 1:** Baseline demographic and clinical characteristics of the study population (*N* = 238).

Characteristic	Value: mean ± SEM or *n* (%).
Age, years	65.1 ± 0.6
Male, *n* (%)	143 (60.1%)
Positive family history of CVD, *n* (%)	145 (61.0%)
BMI, kg/m^2^	28.6 ± 0.3
BMI ≥ 30 kg/m^2^ (Obesity), *n* (%)	80 (33.6%)
T2DM, *n* (%)	93 (39.1%)
Prior MI, *n* (%)	99 (41.6%)
Prior stroke, *n* (%)	24 (10.1%)
Statin therapy	
None, *n* (%)	64 (26.9%)
Atorvastatin 10–20 mg, n (%)	41 (17.2%)
Atorvastatin 40 mg, *n* (%)	78 (32.8%)
Rosuvastatin 20 mg, *n* (%)	52 (21.8%)
Rosuvastatin 40 mg, *n* (%)	3 (1.3%)
Ethnicity	
Kazakh, *n* (%)	120 (50.4%)
Russian, *n* (%)	68 (28.6%)
Uyghur, *n* (%)	25 (10.5%)
Other, *n* (%)	25 (10.5%)

SEM, standard error of the mean, CVD, cardiovascular disease, BMI, body mass index, T2DM, type 2 diabetes mellitus, prior MI, prior myocardial infarction.

Regarding lipid-lowering therapy, approximately one-third of patients (27%, *n* = 64) were not receiving statins at admission. Among those on treatment, high-intensity statin therapy predominated: atorvastatin 40 mg (32.8%), rosuvastatin 20 mg (21.8%), and rosuvastatin 40 mg (1.3%), while 17.2% were receiving low-dose atorvastatin (10–20 mg).

### Lipid levels and Lp(a) distribution

**Figure 1:**
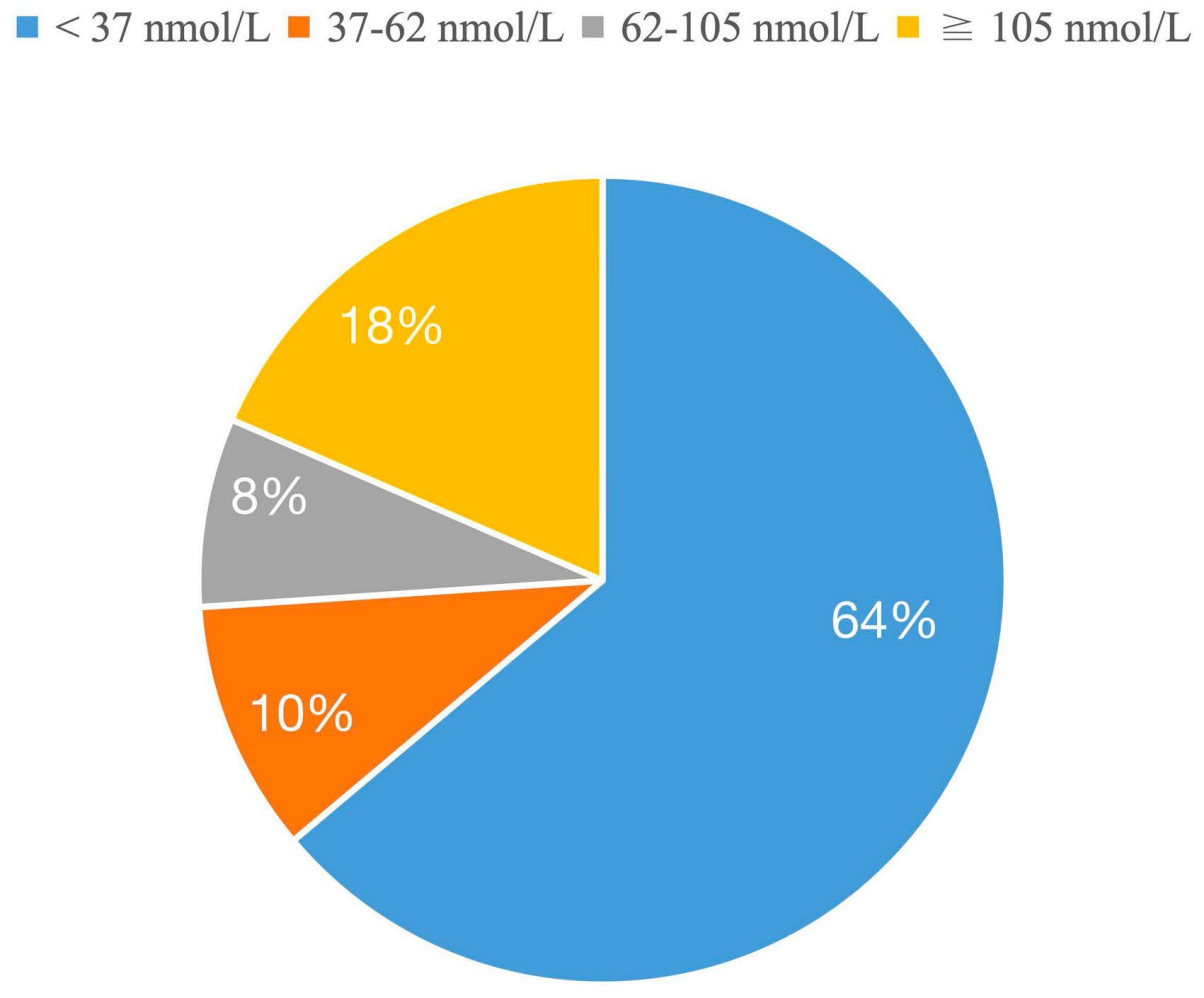
The distribution of patients by Lp(a) concentration categories.

In this cohort, the mean LDL-C was 2.5 ± 0.07 mmol/L, and only 13% (n = 31) achieved the guideline-recommended target of <1.4 mmol/L. Among patients with prior MI, goal attainment was similarly low (13%, 13/99), with a comparable mean LDL-C of 2.5 ± 0.1 mmol/L. The mean plasma Lp(a) concentration in the cohort was 63.2 ± 6.3 nmol/L, with substantial inter-individual variability (Figure 1). Most patients (74%) had Lp(a) levels < 62 nmol/L (<30 mg/dL; generally considered low risk), while 18% had levels > 105 nmol/L (≈50 mg/dL; markedly elevated). The remaining 8% had intermediate Lp(a) concentrations (62–105 nmol/L).

Patients with a prior MI demonstrated significantly higher Lp(a) concentrations than those without prior MI. The mean Lp(a) among post-MI patients was 78.9 ± 11.9 nmol/L, approximately 1.5 times higher than the mean of 51.9 ± 6.6 nmol/L in patients without an MI history (*p* = 0.04). By contrast, conventional lipid measures did not differ significantly between those with and without prior MI. Both groups had similar mean total cholesterol, LDL-C, and high-density lipoprotein cholesterol (HDL-C) levels (Table 2). This suggests that Lp(a) was the distinguishing lipid factor associated with a history of MI in our sample.

**Table 2:** Lipid profile by MI history and angiographic CAD severity.

Group	Lp(a) (nmol/L)	TC (mmol/L)	LDL-C (mmol/L)	HDL-C (mmol/L)
All patients (*N* = 238)	63.2 ± 6.3	4.2 ± 0.08	2.5 ± 0.07	1.2 ± 0.03
Prior MI (*n* = 99)	78.9 ± 11.9*	4.3 ± 0.1	2.5 ± 0.1	1.1 ± 0.04
No prior MI (*n* = 139)	51.9 ± 6.6	4.4 ± 0.2	2.5 ± 0.09	1.2 ± 0.04
No significant stenosis (*n* = 53)	36.5 ± 9.4	4.4 ± 0.1	2.7 ± 0.1	1.2 ± 0.2
1-vessel disease (*n* = 73)	45.3 ± 9.0	4.4 ± 0.1	2.5 ± 0.1	1.2 ± 0.1
2-vessel disease (*n* = 53)	76.7 ± 14.6 *	4.3 ± 0.3	2.4 ± 0.1	1.1 ± 0.1
≥3-vessel disease (*n* = 59)	97.2 ± 15.8 **	4.4 ± 0.2	2.4 ± 0.1	1.2 ± 0.04

MI, myocardial infarction; CAD, coronary artery disease; Lp(a), lipoprotein (a); TC, total cholesterol; LDL-C-low-density lipoprotein cholesterol; (HDL-C), high-density lipoprotein cholesterol.

Values are presented as mean ± SEM. *p < 0.05 and **p < 0.01 for comparisons of Lp(a) levels versus the reference group (no stenosis). No significant differences were observed in total cholesterol, LDL-C, or HDL-C across CAD severity categories.

### Coronary angiography findings

Coronary angiography demonstrated that 53 patients (22%) had no significant epicardial coronary stenosis (non-obstructive CAD), despite a clinical diagnosis of ischemic heart disease, likely reflecting microvascular angina or false-positive functional testing. Angiographically significant CAD, defined as ≥50% stenosis in at least one major epicardial artery, was identified in the remaining 185 patients (78%). Disease severity was distributed as follows: single-vessel disease in 73 patients (30.7%), two-vessel disease in 53 patients (22.3%), and three-vessel or multivessel disease in 59 patients (24.8%).

**Figure 2:**
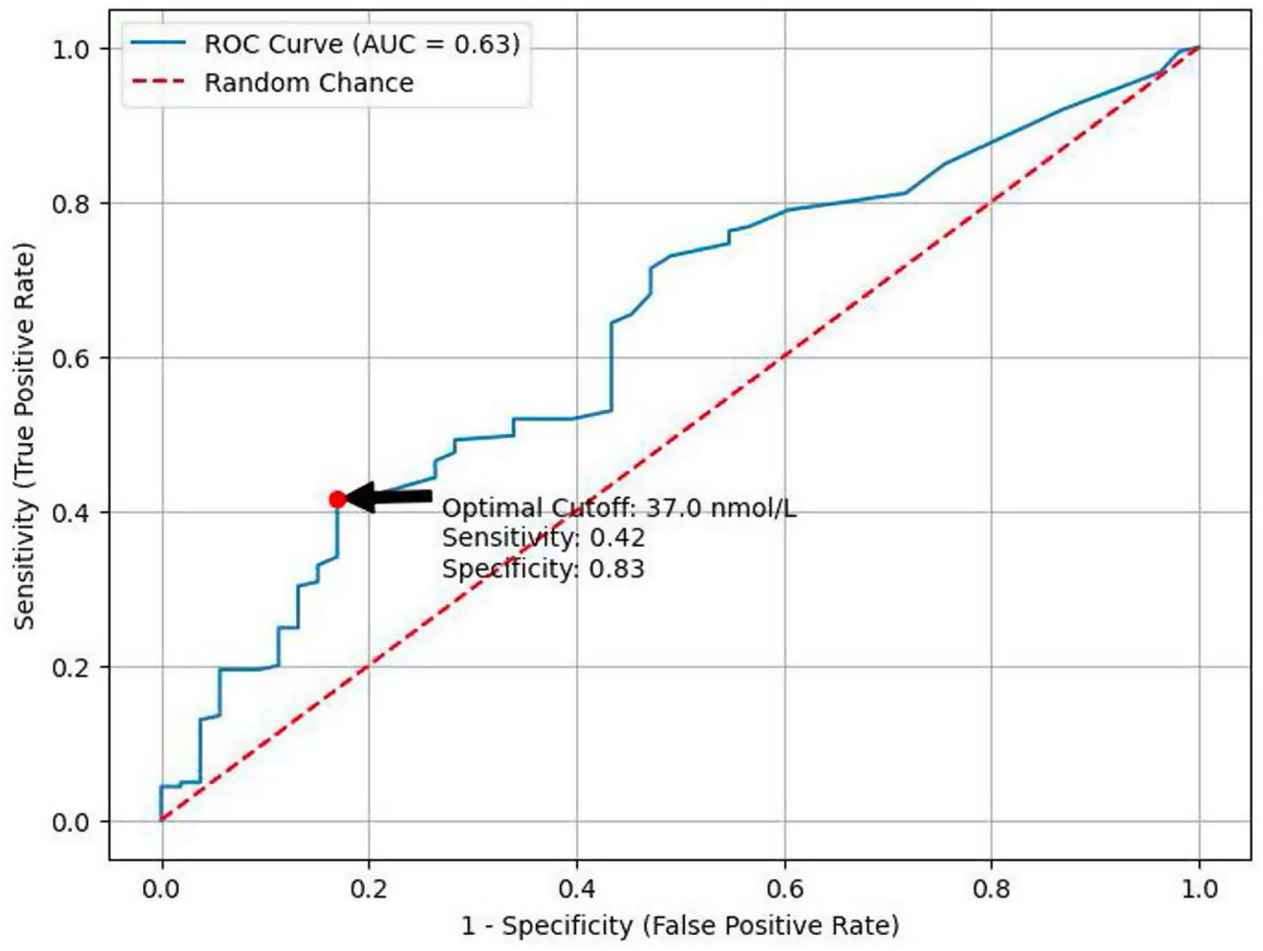
ROC analysis of Lp(a) for detection of significant CAD. ROC, receiver operating characteristic; AUC, area under the curve.

A clear and graded association was observed between Lp(a) concentrations and the angiographic extent of CAD. Patients without significant stenosis had the lowest Lp(a) levels (36.5 ± 9.4 nmol/L) (Figure 2). Lp(a) concentrations increased progressively with disease severity, reaching 45.3 ± 9.0 nmol/L in single-vessel disease, 76.7 ± 14.6 nmol/L in two-vessel disease, and 97.2 ± 15.8 nmol/L in three-vessel or multivessel CAD. These differences were statistically significant (*p* < 0.05 for two-vessel vs. no stenosis; *p* < 0.01 for ≥3-vessel vs. no stenosis; Table 2).

In contrast, conventional lipid parameters—including total cholesterol, LDL-C, and HDL-C—did not differ significantly across CAD severity categories. Thus, Lp(a) emerged as the only lipid parameter that consistently tracked with the presence and extent of coronary atherosclerosis in this cohort.

**Figure 3:**
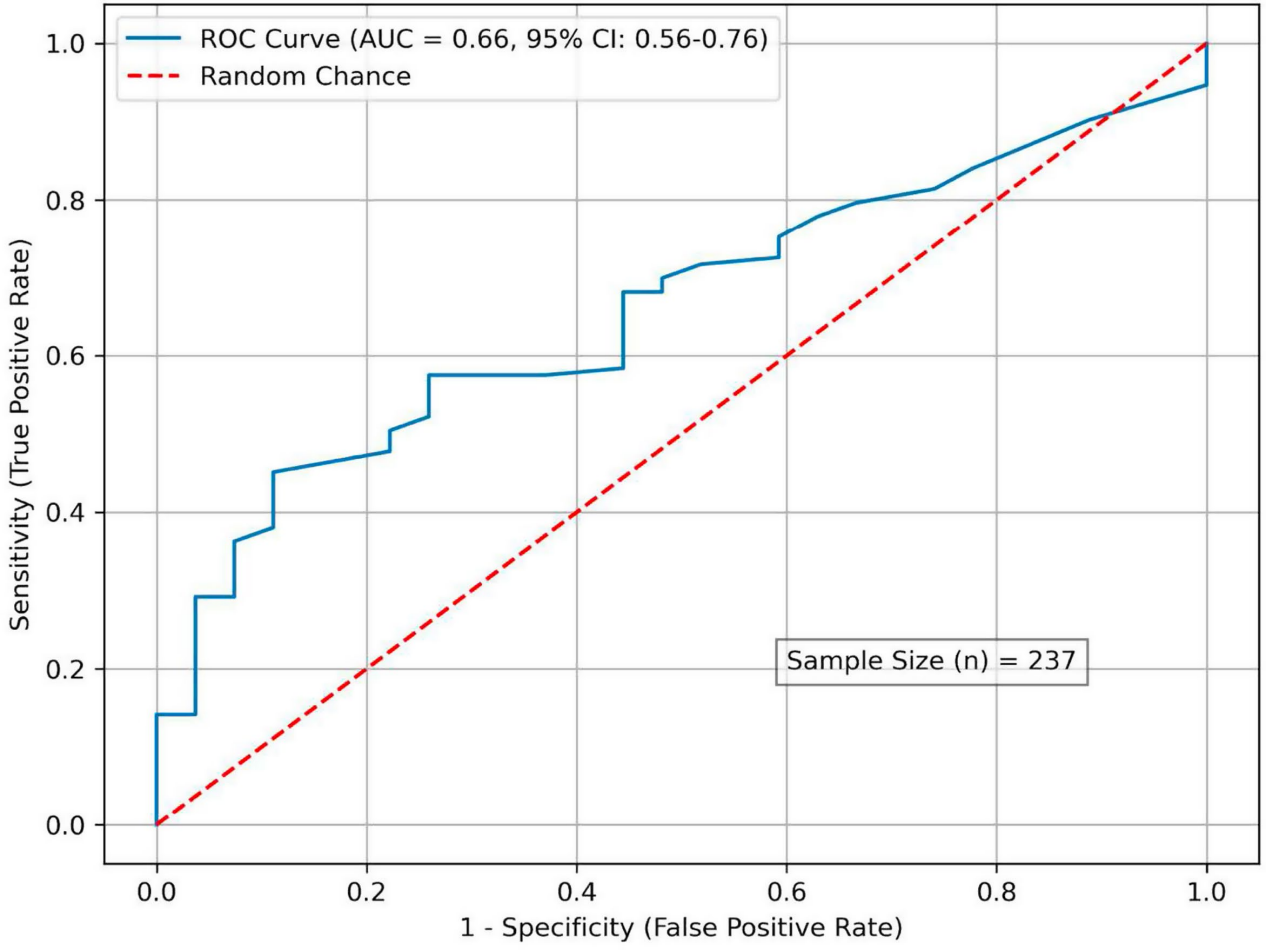
ROC analysis: Lp(a) levels for stenosis prediction. AUC, area under curve.

Receiver operating characteristic (ROC) curve analysis was performed to identify an Lp(a) threshold associated with angiographically significant CAD. The area under the curve (AUC) was 0.660 (95% CI 0.558–0.757), indicating modest but clinically meaningful discriminative ability (Figure 3). The optimal cut-off, determined using the Youden index, was 37.0 nmol/L, yielding a specificity of 83% and a sensitivity of 42% for detecting obstructive CAD. Patients with Lp(a) ≥ 37 nmol/L had a significantly higher prevalence of angiographic CAD compared with those below this threshold (90% vs. 71%; *p* < 0.001), corresponding to an odds ratio of 3.4 (95% CI 1.6–7.6; Table 3).

**Table 3:** Association of Lp(a) with the presence of significant coronary artery disease.

Angiographic severity of CAD	Lp(a) ≤ 37 nmol/L, *n* (%)	Lp(a) ≥ 37 nmol/L, *n* (%)	*p*-value	OR (95%CI)
Non- significant CAD	44 (29%)	9 (10%)	0.001	Reference
Significant CAD (≥1 vessel disease)	109 (71%)	76 (90%)	0.001	3.4(1.6–7.6)

Coronary artery disease - (CAD) presence versus Lp(a) threshold (37 nmol/L). Patients are divided by Lp(a) > 37 nmol/L vs. ≤ 37 nmol/L. CAD defined as ≥1 significant coronary stenosis on angiography.

OR = odds ratio for having CAD with high Lp(a) vs. low Lp(a).

Overall, these findings demonstrate that even moderately elevated Lp(a) levels—well below commonly used Western thresholds—are strongly associated with both the presence and severity of coronary atherosclerosis in this population, independent of traditional lipid parameters.

### Multivariable analysis

To assess whether the association between Lp(a) and CAD severity was independent of traditional cardiovascular risk factors, a multivariate logistic regression analysis was performed with the presence of angiographically significant stenosis (≥50% in ≥1 epicardial vessel) as the dependent variable. The model included age, diabetes mellitus, body mass index, family history of premature cardiovascular disease. The overall model was statistically significant (likelihood ratio test *p* = 0.025). Importantly, Lp(a) remained a strong independent predictor of significant coronary stenosis (*β* = 0.006, *p* = 0.004), indicating an increasing probability of obstructive CAD with rising Lp(a) concentrations (Table 4). Diabetes mellitus (OR ≈ 2.10, *p* = 0.044) and a positive family history of premature cardiovascular disease (OR ≈ 1.74, *p* = 0.011) were also independently associated with CAD presence. These findings confirm that the relationship between Lp(a) and CAD severity persists after adjustment for established clinical risk factors.

**Table 4:** Multivariable logistic regression analysis for the presence of significant coronary artery disease.

Variables	Coeff	Standard error	*p*-value
Diabetes Meliitus	0.7418	0.369	0.044
Lp(a)	0.0060	0.002	0.004
Family history	0.5561	0.218	0.011

## Discussion

Our findings reinforce that elevated lipoprotein(a) [Lp(a)] concentration is an independent determinant of coronary atherosclerosis severity, even in a population with substantial statin use. Patients with higher Lp(a) levels exhibited more extensive angiographic coronary artery disease (CAD) and were more likely to have a history of myocardial infarction. These observations align with a growing body of evidence demonstrating that Lp(a) contributes meaningfully to residual cardiovascular risk, particularly in settings where LDL-C is partially or adequately controlled (14).

In the present cohort, 18% of patients had Lp(a) concentrations >105 nmol/L (50 mg/dL), consistent with the approximately 20% global prevalence of elevated Lp(a) (2). Conversely, nearly three-quarters of participants had Lp(a) levels <62 nmol/L (30 mg/dL), a distribution comparable to that reported in other population studies. A key insight from our data, however, is that the absolute Lp(a) concentrations associated with clinically significant CAD were substantially lower than the thresholds commonly applied in Western populations. Receiver operating characteristic (ROC) analysis identified a threshold of approximately 37 nmol/L associated with obstructive CAD; however, the discriminative performance was modest (AUC 0.66) with limited sensitivity (42%). These findings indicate that this threshold should not be interpreted as a standalone diagnostic cut-off, but rather as a marker of increased cardiovascular risk, best applied in conjunction with established clinical and biochemical risk factors.

From a clinical perspective, Lp(a) measurement represents a one-time, genetically determined biomarker, which supports its cost-effectiveness even in resource-limited settings. Our findings suggest that even modest elevations (≥37 nmol/L in this cohort) may warrant intensification of overall cardiovascular risk management. In the absence of widely available Lp(a)-lowering therapies, the primary clinical utility of Lp(a) lies in risk reclassification and the prioritization of preventive strategies, including more aggressive control of modifiable risk factors. Importantly, Lp(a) assessment can be readily incorporated into routine lipid evaluation without significant additional burden, facilitating its implementation in everyday clinical practice.

Current Western guidelines typically define Lp(a) levels of 30 mg/dL and 50 mg/dL as moderate- and high-risk cut-offs, respectively (2), whereas Chinese guidelines classify >30 mg/dL as high risk due to an earlier risk inflection (11). In our study, the mean Lp(a) concentration among patients with prior myocardial infarction was approximately 79 nmol/L (~31.5 mg/dL), well below the 50 mg/dL threshold, yet clearly associated with significant clinical disease. These findings are consistent with previous studies from Kazakhstan: Telemtaeva et al. reported mean Lp(a) levels of ~26.5 mg/dL in patients with myocardial infarction (15), while Bekbossynova et al. observed increased ASCVD risk beginning at approximately 21.2 mg/dL (12). Our identified threshold for predicting obstructive CAD (~37 nmol/L, ~18 mg/dL) aligns with this emerging regional pattern. However, its clinical interpretation warrants caution. Although ROC analysis identified this level as the optimal cut-off, the overall discriminative performance was modest (AUC 0.66) with limited sensitivity (42%), indicating that it should not be used as a standalone diagnostic threshold. Rather, Lp(a) at this level should be considered a marker of increased cardiovascular risk and interpreted in conjunction with established clinical and biochemical factors. Collectively, these findings suggest that internationally accepted Lp(a) cut-offs may be too high for Central Asian populations, where even relatively modest elevations appear to confer substantial cardiovascular risk.

The absence of an association between raw LDL-C and angiographic disease burden in our cohort may be explained by the widespread use of statin therapy. While statins effectively reduce circulating LDL-C levels, they have little to no effect on Lp(a) concentrations and, in some studies, may even modestly increase Lp(a) levels (2, 16). As a result, LDL-C–mediated risk may be attenuated in statin-treated patients, whereas Lp(a)-related risk persists and remains clinically evident (2, 17). This dissociation is clearly reflected in our data: LDL-C did not correlate with CAD severity, while Lp(a) demonstrated a strong and graded association with both the presence and extent of angiographic atherosclerosis. These findings reinforce the concept of Lp(a) as a distinct and independent driver of residual cardiovascular risk, operating through pathways not addressed by standard lipid-lowering therapy (1, 2, 17).

Ethnic differences in Lp(a) biology are likely to contribute to the observed variation in risk thresholds. Up to 90% of interindividual variability in Lp(a) concentrations is genetically determined by the LPA locus (16, 18, 19), with an additional 40–70% attributable to apolipoprotein(a) isoform size, which shows a strong inverse relationship with circulating Lp(a) levels (16, 20). Individuals carrying low–molecular-weight apo(a) isoforms (10–22 kringle IV repeats) exhibit Lp(a) concentrations approximately 4–5 times higher than those with high–molecular-weight isoforms (16). Large population studies, including the Dallas Heart Study, have demonstrated a linear association between kringle IV repeat number and Lp(a) concentrations across ethnic groups, although individuals of African ancestry consistently display higher Lp(a) levels at any given isoform length (21). Comparable ethnicity-specific patterns may exist in Central Asian populations but remain insufficiently characterized.

The present study reflects the ethnic diversity of Kazakhstan, with a cohort comprising predominantly Kazakh, Russian, and Uyghur participants. Although the study was not powered to derive ethnicity-specific diagnostic thresholds, the association between elevated Lp(a) levels and coronary atherosclerosis was consistent across major ethnic subgroups. These findings support the ethno-specific premise of the study and provide a strong rationale for future, adequately powered investigations aimed at refining population- and ethnicity-specific Lp(a) risk thresholds in Central Asia.

Our results are also concordant with data from other populations demonstrating that elevated Lp(a) is associated not only with the presence but also with the severity and diffuseness of coronary atherosclerosis. Leistner et al. showed that high Lp(a) levels were linked to more complex CAD phenotypes, including higher SYNTAX and Gensini scores and a greater prevalence of chronic total occlusions, independent of LDL-C (14, 17). Similarly, in our cohort, patients with the most severe multivessel disease had mean Lp(a) levels of approximately 97 nmol/L (~39 mg/dL)—values that might be considered only mildly elevated by Western standards, yet were clearly associated with extensive plaque burden.

Mechanistically, Lp(a) promotes atherothrombosis through multiple pathways. It serves as a carrier of oxidized phospholipids that enhance vascular inflammation, and apolipoprotein(a) shares structural homology with plasminogen, impairing fibrinolysis and fostering a pro-thrombotic milieu. These properties likely contribute to both atherosclerotic plaque development and thrombotic complications. The lack of any relationship between LDL-C and CAD severity in our study further underscores that Lp(a) represents a distinct pathogenic axis, separate from traditional lipid parameters. While statins likely reduced LDL-driven risk in this cohort, Lp(a) remained unmodified by standard therapy and emerged as the principal lipid marker associated with disease burden.

Taken together, these observations emphasize that Central Asian individuals with even moderately elevated Lp(a) concentrations should be identified and monitored more closely. Until targeted Lp(a)-lowering therapies become widely available, management strategies should focus on aggressive control of modifiable risk factors—including intensive LDL-C reduction, optimal blood pressure control, and lifestyle interventions—to mitigate the excess risk associated with elevated Lp(a).

In summary, this study demonstrates that in a Kazakhstani population, Lp(a) is a strong marker of coronary artery disease burden, with clinically meaningful risk manifesting at lower absolute levels than traditionally recognized. These findings support the need to adapt Lp(a)-related risk thresholds to specific ethnic and regional contexts and to incorporate routine Lp(a) measurement into cardiovascular risk assessment in Central Asia. Larger prospective studies are warranted to refine population-specific thresholds and to determine whether emerging Lp(a)-lowering therapies will translate into improved clinical outcomes in this region.

## Data Availability

The raw data supporting the conclusions of this article will be made available by the authors, without undue reservation.
